# Novel Biofuel Cell Using Hydrogen Generation of Photosynthesis

**DOI:** 10.3390/jfb11040081

**Published:** 2020-11-11

**Authors:** Akinari Iwahashi, Takuya Yamada, Yasumitsu Matsuo, Hinako Kawakami

**Affiliations:** Faculty of Science & Engineering, Setsunan University, Ikeda-nakamachi, Neyagawa, Osaka 572-8508, Japan; 20m901ia@edu.setsunan.ac.jp (A.I.); t-yamada@rikengreen.co.jp (T.Y.); kawakami@led.setsunan.ac.jp (H.K.)

**Keywords:** photosynthesis, hydrogen source, fuel cell, photosystem II

## Abstract

Energies based on biomaterials attract a lot of interest as next-generation energy because biomaterials are environmentally friendly materials and abundant in nature. Fuel cells are also known as the clean and important next-generation source of energy. In the present study, to develop the fuel cell based on biomaterials, a novel biofuel cell, which consists of collagen electrolyte and the hydrogen fuel generated from photochemical system II (PSII) in photosynthesis, has been fabricated, and its property has been investigated. It was found that the PSII solution, in which PSII was extracted from the thylakoid membrane using a surfactant, generates hydrogen by the irradiation of light. The typical hydrogen-generating rate is approximately 7.41 × 10^14^ molecules/s for the light intensity of 0.5 mW/cm^2^ for the PSII solution of 5 mL. The biofuel cell using the PSII solution as the fuel exhibited approximately 0.12 mW/cm^2^. This result indicates that the fuel cell using the collagen electrolyte and the hydrogen fuel generated from PSII solution becomes the new type of biofuel cell and will lead to the development of the next-generation energy.

## 1. Introduction

It is well known that clean and environmentally friendly energies are strongly desired as the next generation of energy. Hydrogen energy is especially emphasized as the next-generation clean energy because CO_2_ emissions are remarkably reduced for producing energy [1,2,3]. However, it is also known that hydrogen gases are commonly produced from the exhaustion of fossil fuels, and therefore, the hydrogen gas production from fossil fuels with high efficiency and hydrogen gas production without fossil fuels is significantly important [4,5,6,7,8,9,10]. For example, Balat et al. reported the importance of biomass-based hydrogen from political, economic, and environmental aspects [5]. In many cases, hydrogen is converted to energy using the fuel cell. The fuel cell generates energy by the chemical reaction from H_2_ and O_2_ to H_2_O [11]. Therefore, power generation efficiency is extremely high compared with the efficiency of the combustion energy of fossil fuels. Currently, it is known that several fuel cells, such as PEMFC (Proton Exchange Membrane Fuel Cell, PAFC (Phosphoric Acid Fuel Cell), MCFC (Molten Carbonate Fuel Cell), SOFC (Solid Oxide Fuel Cell), DMFC (Direct Methanol Fuel Cell), etc. [12,13,14,15,16,17,18,19,20,21,22,23]. Rikukawa and Sanui have shown the synthesis, chemical, and electrochemical properties, and the polymer-electrolyte fuel cell applications of new proton-conducting polymer electrolyte membranes based on hydrocarbon polymers [14]. Asensio et al. reported that the proton-conducting membranes based on benzimidazole polymers become high-temperature PEM fuel cells [15]. For the direct methanol fuel cell, Goor et al. reported the fabrication of low-cost and high-power DMFC, and its possibility of mobility and portable applications of DMFC has been shown [19]. Pareta et al. suggested the possibility of the Methanol reformer integrated phosphoric acid fuel cell (PAFC) based power plants [21]. In SOFC, a detailed overview of a lot of SOFC related materials and devices has been summarized by Dwivedi [22]. In this way, there are a lot of reports for the fuel cell and electrolytes.

It is also known that biological materials are beneficial for proton conduction, hydrogen production, etc. For example, biomaterials such as DNA, collagen, chitin, and chitosan show relatively high proton conductivity under humidified conditions and become the electrolyte of the fuel cell [24,25,26,27,28,29,30,31,32,33,34]. Recently, it was reported that a DNA-electrolyte fuel cell was fabricated and that the proton conductivity of DNA electrolyte is caused by forming the water bridges in DNA [24,25,26]. Moreover, it was reported that the collagen film also becomes a proton conductor and that the collagen-electrolyte fuel cell has been fabricated [27,28,29,30]. Furthermore, Freier et al. showed the results of proton transfer via a transient linear water-molecule chain in a membrane protein [31], and Ordinario et al. showed the bulk protonic conductivity in a cephalopod structural protein [32]. In addition, Kawabata et al. fabricated the chitin-based fuel cell, and its proton conductivity was reported [33,34]. Thus, recently, there are a lot of research works on the proton conductor of biomaterials.

Moreover, for the hydrogen production of biomaterials, there are many investigations concerning hydrogen productions such as fermentative reactions, microbial electrolysis cell reactions, enzyme reactions of substrates such as glucose, and reactions by the anaerobic environment in the algae and so on [35,36,37,38,39,40,41,42,43]. For example, Wang and Wan have investigated the effect of temperature on fermentative hydrogen production by mixed cultures and determined the optimal temperature for fermentative hydrogen production [36]. Zhang et al. improved hydrogen production from glucose by adding a specific methane inhibitor to repress the activity of methanogens microbial electrolysis cells [37]. Thus, investigations of hydrogen production using biomaterials are carried out actively.

In addition, there are some investigations for photo-biochemical cells. For example, Yehezkeili et al. have fabricated the poly (mercapto-p-benzoquinone)/photosystem II/bilirubin oxidase/carbon nanotubes photoelectrochemical cell and reported its characteristics [44]. Zhang and Reisner summarized photoelectronchemistry concerning electrodes, protein, and bio-material interface and reported the role of the biological photosynthetic system in semi-artificial photosynthesis [45]. In this way, the investigations concerning the biochemical cells are also carried out with a lot of interests.

**Figure 1:**
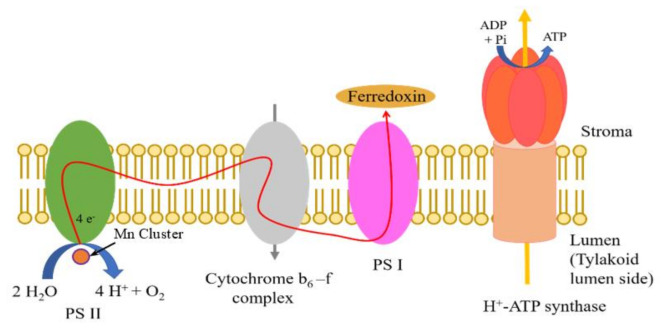
Schematic model of thylakoid membrane.

On the other hand, it is also known that hydrogen ions are used in the process of photosynthesis. Figure 1 shows the schematic model of the thylakoid membrane, which is the central role in photosynthesis. As well-known, in the thylakoid membrane, there exists the membrane protein complex such as photosystem I (PSI) and photosystem II (PSII) concerning the absorption of light, electron and proton transfers, water splitting reaction, and so on [46,47,48]. As shown in Figure 1, light irradiation causes the water-splitting reaction at PSII, and the hydrogen and oxygen ions and electrons are produced. Commonly, the generated electrons are passed to the cytochrome b6-f complex and are used in the reaction of nicotinamide adenine dinucleotide phosphate (NADPH) at PSI. The generated hydrogen ions are passed to adenosine triphosphate (ATP) synthase usually. The deprotonation of the water molecule in PSII is carried out at the manganese (Mn) cluster reported by several articles [49,50,51]. The structure of the Mn cluster resembles the distorted chair, and the bonding of water molecules is distorted. As a result, the deprotonation of the water molecules is achieved with light energy easier. In this way, it is important to obtain the PSII without the break of the Mn cluster. The solubilization of the biological membrane is realized by a surfactant. Miyao, Shen, and Enami reported the PSII extraction using the surfactant [52,53]. Therefore, by controlling the surfactant composition, PSII can be separated from the thylakoid membrane. In this way, if we can separate PSII from the thylakoid membrane, we can extract the hydrogen ion from the water-splitting reaction by the light irradiation. However, the actual energy source using the hydrogen production by the light irradiation to PSII solution has not been realized yet, although several prospections were reported [42,43]. Especially, there are no results that hydrogens extracted by separating the PSII from the thylakoid membrane as the PSII solution are directly applied to the fuel of fuel cells based on bioelectrolyte, although the possibility for hydrogen production such as ferredoxin, reduced ferredoxin (Fd), and a reverse hydrogenase was speculated. In the present work, we explored the possibility of the hydrogen generation from the PSII solution and fabricated the PSII biofuel cell using hydrogens generated by the irradiation to the PSII solution. This work will be helpful in facilitating the investigation of new hydrogen-energy sources in the field of environmentally friendly energy.

## 2. Materials and Methods

### 2.1. Bioelectrolyte “Collagen”

**Figure 2:**
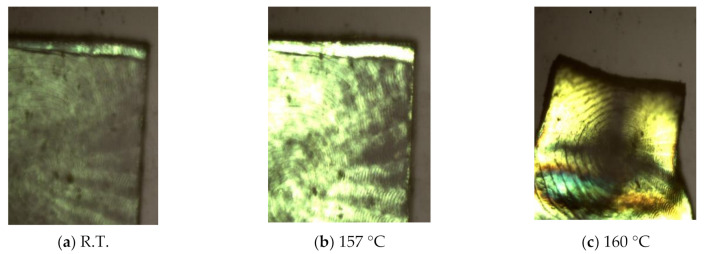
Photograph of collagen film observed at various temperatures: (**a**) R.T.; (**b**) 157 °C; (**c**) 160 °C.

In the present study, collagen films were used as electrolytes of the fuel cell. The collagen films, which were extracted from the decalcified fish scales of Tilapia fish, were provided by Nitta Gelatin Inc. In the previous paper, we found that the collagen film becomes a proton conductor under the humidified condition [28,29]. Moreover, it was also known that the collagen film becomes the electrolyte of the fuel cell [27,29]. Figure 2a shows the photograph of the collagen film observed under a polarized microscope at room temperature [30]. As shown in Figure 2a, the collagen films are semitransparent. The thickness of collagen films used as fuel cell electrolytes was adjusted to approximately 135 μm. Figure 2b,c show the photograph of collagen films observed at 157 °C and 160 °C, respectively. It is evident that the collagen films were stable below 157 °C, although they became softening above 160 °C. In this way, the collagen films obtained from the fish scale have excellent thermal stability under 160 °C. From these results, it is apparent that collagen film obtained from the fish scale possesses the durability and that therefore the collagen films can be used as the electrolyte of the fuel cell without a problem.

**Figure 3:**
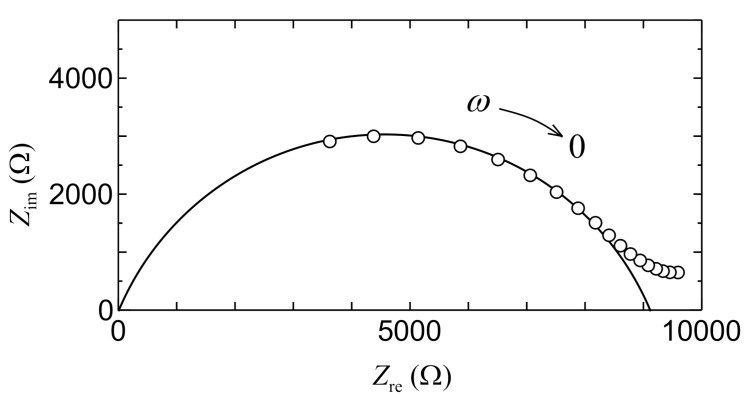
Relation between *Z*_re_ and *Z*_im_ in collagen electrolyte.

Figure 3 shows the relation between the real part *Z*_re_ and the imaginary part *Z*_im_ of the impedance of the collagen used as the fuel cell electrolyte. The impedance measurement was carried out with the precision LCR meter (E4980A, Agilent Technologies Japan, Ltd., Tokyo, Japan) in the humidified condition at room temperature. The thickness of collagen films used in this measurement was adjusted to approximately 135 μm. The area of electrodes is 0.159 cm^2^. As shown in Figure 3, the relation between *Z*_re_ and *Z*_im_ becomes a semi-circle one. This result indicates that the collagen electrolyte is represented by the parallel equivalent circuit of resistance *R* and capacitance *C*. In this case, *Z*_re_ and *Z*_im_ are shown in the following equations:

(1) $$Z_{\mathrm{re}}=\frac{R}{1+{\left(\omega CR\right)}^{2}},Z_{\mathrm{im}}=\frac{\omega CR^{2}}{1+{\left(\omega CR\right)}^{2}}$$

here, *ω* is the angular frequency.

When *ω* → 0, *Z*_re_ = *R* and *Z*_im_ = 0. That is, we can obtain bulk DC proton conductivity of the collagen electrolyte from *Z*_re_ extrapolated to *ω* → 0, considering that the measured impedance for low angular frequency mainly results from the double layer capacitance near electrodes. By using Figure 3, proton conductivity used as the bioelectrolyte is calculated to be 0.93 × 10^−5^ S/cm. This value is consistent with the value of ~1.0 × 10^−5^ S/cm of the proton conductivity obtained from other measurements in the high humidified condition [29]. The proton conductivity of the solid collagen electrolyte is caused by the proton (or H_3_O^+^) migration via the water bridges formed between OH, CO, and NH groups in the side chain of the collagen peptide [29]. Due to the simple mechanism of a water bridge without the doping of active group such as SO_3_H, the proton conductivity (~1 × 10^−5^ S/cm) in the collagen film is lower compared with the other proton conductors such as Nafion (~1 × 10^−1^ S/cm) including SO_3_H [54] and the hydrogel (~1 × 10^−3^ S/cm) based on PVA and buffer solutions [55]. However, fortunately, the low proton conductivity of the collagen film suits the present PSII-biofuel cell system because the hydrogen generated from the PSII solution of 5 mL is not much.

### 2.2. Preparation of PSII Containing Solution

**Figure 4:**
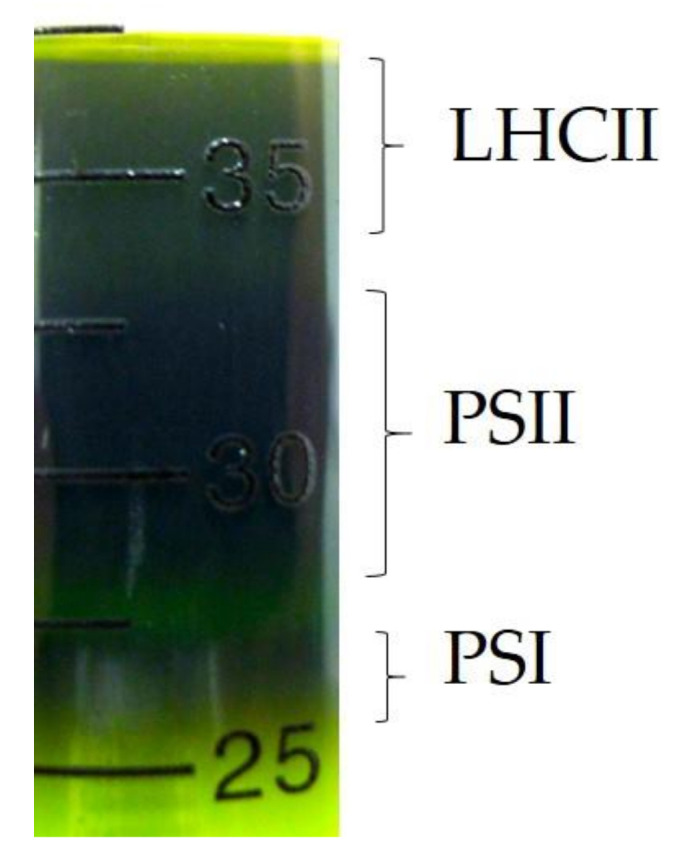
Photograph of sucrose density gradient in the PSII containing solution.

The PSII containing solution (hereinafter called “the PSII solution”) was extracted from the spinach leaves. The extraction of the PSII solution was carried out at 4 °C in the dark. First, the spinach leaves were crushed in a distilled water and were filtrated. The filtrated solution, green in color, was centrifuged at 6000 rpm for 15 min. Next, the obtained precipitate was suspended in the distilled water. The obtained solution was centrifuged at 6000 rpm for 15 min again, and the supernatant was removed. The obtained precipitate was suspended in the distilled water to satisfy the condition of 2 mg/mL. This extracted solution was solved by the 20 *w*/*v*% nonionic surfactant (Triton x-100: Nacalai tesque Inc., Kyoto, Japan) diluted with the distilled water. Further, this PSII containing solution was centrifuged at rpm for 60 min, and the precipitate was extracted in order to eliminate the PSI complex, which was the lightweight compared with the PSII complex. Finally, the obtained precipitate was suspended in distilled water, and its solution was centrifuged twice at 12,000 rpm for 60 min, and after that the PSII solution was extracted. The presence of the PSII solution was confirmed by the sucrose density gradient. The result is shown in Figure 4. As shown in Figure 4, the gel bands of light-harvesting chlorophyll protein complex II (LHCII), PSII core, and PSI–light harvesting complex I (PSI-LHCI) complexes are observed, similar to Figure 1 of Ref. [56]. In this way, the PSII solution used in the present work includes a lot of the PSII complex. The obtained PSII solution was introduced to the anode of the fuel cell and the output characteristics of the PSII biofuel cell using the PSII solution as the fuels were investigated.

### 2.3. Measurement of Hydrogen Gas Concentration

Hydrogen gas generated from the PSII solution was collected inside the polyethylene terephthalate (PET) bag. The H_2_ gas permeability of PET film is known to be extremely low [57]. The calibrated hydrogen gas sensor (MQ-8; Hanwei Electronics Co., Ltd., Zhengzhou, China) was settled in the PET bag, and the air of 1.0 L was purged. Then, the gas outlet of the PSII solution was connected with the PET bag without the leakage of gas, and the output from the sensor was obtained as a ppm unit. We were able to obtain the hydrogen molecular number from the amount of the purge gas and the hydrogen concentration obtained with ppm unit. In this way, the hydrogen molecular number generated by the PSII solution was measured.

### 2.4. Preparation of PSII Biofuel Cell Using Bioelectrolyte

**Figure 5:**
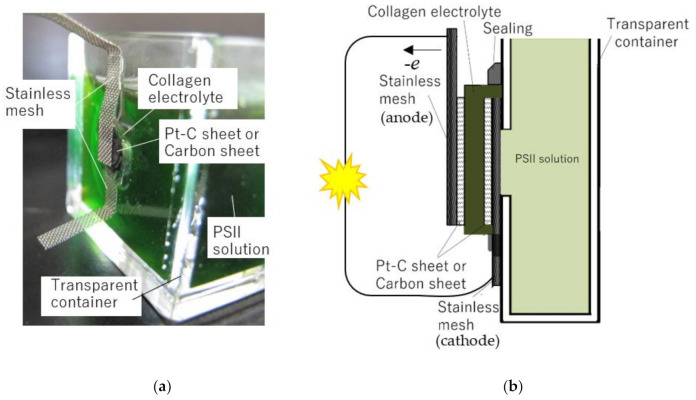
PSII biofuel cell (**a**) photograph and (**b**) schematic diagram.

Figure 5 shows the photograph of the PSII biofuel cell and a schematic diagram of the biofuel cell using the PSII solution as a fuel of the PSII biofuel cell. As shown in Figure 5a, the PSII biofuel cell consists of the PSII solution, transparent container, stainless mesh plates, Pt-C catalysts, and the bioelectrolyte. In the present work, the collagen film was used as the bioelectrolyte. The thickness of the collagen film was typically 135 μm. The collagen film was sandwiched between an anode and a cathode. The electrodes of the cathode and the anode constructed by the carbon sheet of 4.5 mm diameter. The collagen film and these electrodes were bonded using the ethanol solution containing Pt-C powder, and a three-phase interface was realized. Here, the ethanol solutions containing the Pt-C powder were made with a concentration of 1 g/mL. In order to collect electrons generated at the anode of the fuel cell, the electron-collecting electrode, which consisted of a stainless mesh of 100 mesh/cm^2^, was mounted on the electrodes. The generated electrons at the anode pass through the external circuit and arrive at the cathode. At the cathode, electrons bond to the oxygen in air and hydrogen ion via the electrolyte, and then H_2_O is produced. In this process, we can obtain the energy as electricity from the fuel cell.

In order to obtain this energy from the fuel cell, the hydrogen ion and oxygen gas must be supplied to the anode and the cathode, respectively. Commonly, the hydrogen and oxygen gases are used as fuel gases of the fuel cell. In the present work, oxygen fuel gases were directly introduced to the cathode from the air. On the other hand, the hydrogen ions were introduced to the anode electrode from the proton source of the PSII solution. That is, the anode electrode and bio-electrolyte contacted the PSII solution, and a three-phase interface was formed. The PSII solution was injected into a transparent container. A hole 5 mm in diameter is made at a part of the container and pasted the biofuel cell. The PSII solution contacts with the anode electrode. To prevent the leakage of the PSII solution, transparent sealing material was used. When the light incidents the PSII solution, the hydrogen ions are generated in the PSII solution. The obtained hydrogen ions at the anode pass through the collagen electrolyte and react at the cathode with oxygen in the air. On the other hand, the produced electrons pass through external circuits, as shown in Figure 5b.

### 2.5. Measurement of Fuel Cell Characteristics

The relation between current density versus cell voltage in the PSII biofuel cell was measured using the potentio-galvanostat (SI1287, Solatron analytical Ltd., Hampshire, UK) and the electronic voltmeter (keithley2100 and 2000, Keithley Instruments Ltd., Solon, OH, USA). The current density *i* flowing through the load was controlled by the potentio-galvanostat and the computer, and the cell voltage *V* was acquired by the computer. The cell temperature was measured using the copper-constantan thermocouple and the electronic voltmeter (Keithley2100), and the light intensity was measured by the electronic voltmeter (Keithley2000), the photodiode (S1722-02, Hamamatsu Photonics K. K., Hamamatsu, Japan) and its current-voltage converter (T-IVA001, Turtle Industry Co., Ltd., Ibaraki, Japan). The cell temperature and light intensity were controlled to be 27 °C and 0.5 mW/cm^2^ respectively, and were captured by the data acquisition system using the computer together with the acquisition of *V*. In the measurement of current density *i*, the volume of PSII solution was adjusted to 5 mL. The relation between *i* and power density *P* in the PSII biofuel cell was also calculated by the data of the *i–V* characteristics with the relation of *P* = *iV*.

## 3. Results

**Figure 6:**
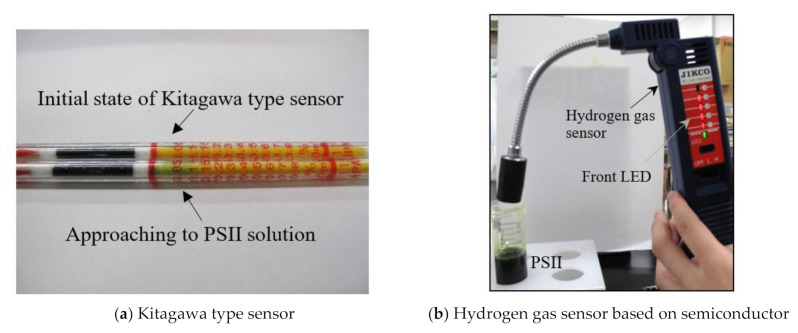
Evidence of hydrogen gas generation. (**a**) Kitagawa type sensor; (**b**) Hydrogen gas sensor based on semiconductor.

Figure 6 shows the photographs of the evidence for hydrogen gas generation using two methods. One is the Kitagawa type sensor (137U Hydrogen, Komyo Rikagaku Kogyo K. K., Kanagawa, Japan), and the other is the hydrogen gas sensor based on a semiconductor (JKC-HY, Ichinen Jikco Co., Ltd., Tokyo, Japan). In the Kitagawa type sensor of Figure 6a, the color of reagent changes from yellow to blue by the following reactions:

H_2_ + 1/2O_2_ → H_2_O, and H_2_O + Mg(ClO_4_)_2_ → Mg(ClO_4_)_2_^·^H_2_O (2)

**Figure 7:**
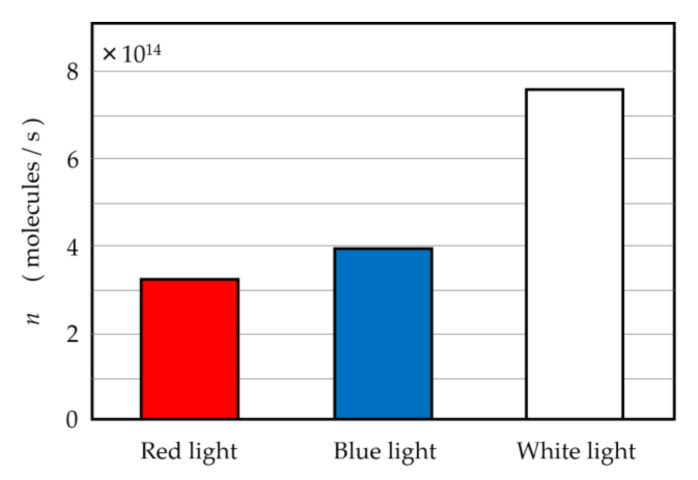
Number of the generated hydrogen gas by the irradiations of the red, blue, and white lights.

In the semiconductor type hydrogen sensor of Figure 6b, the LED lights on the front of the sensor turn on by the existence of hydrogen gas. As shown in Figure 6a, the reagent color of the Kitagawa type sensor changes from yellow to green by approaching the PSII solution. In addition, as shown in Figure 6b, the red LED lamps on the front of the hydrogen gas sensor based on semiconductor turn on by approaching the PSII solution. These results indicate that the hydrogen gas is actually generated from the PSII solution. In this way, the PSII solution can produce hydrogen gas by the irradiation of light. In order to investigate the hydrogen-gas generation of the PSII solution quantitatively, we measured the generated hydrogen molecular number per second, *n*, by the irradiation of blue (wavelength 440 nm), red (wavelength 650 nm), and white lights to the PSII solution using the hydrogen gas sensor based on the semiconductor. These results are shown in Figure 7. The integrated irradiance of blue and red lights is 0.5 mW/cm^2^, and the integrated irradiance of white light is also controlled to be 0.5 mW/cm^2^.

As shown in Figure 7, the hydrogen gas was generated by all the irradiations of blue, red, and white lights. The values of the generated hydrogen molecular number per second for the blue and red light irradiations were 3.95 × 10^14^ and 3.28 × 10^14^ for the PSII solution of 5 mL, respectively. For the irradiation of white light, *n* became 7.61 × 10^14^ molecules/s for the PSII solution of 5 mL. We can estimate the maximum current *i*_0_ from *n* using the Faraday’s second law of electrolysis “*i*_0_ = *znF/N*_A_”, where *z* and *n* are the total charge number of the hydrogen molecule, and the hydrogen molecular number per unit time, respectively, and *F* and *N*_A_ are the Faraday constant and Avogadro constant, respectively. The maximum current obtained from Faraday’s law is 0.24 mA using *z* = 2 and *n* = 7.61 × 10^14^ molecules/s. This value is consistent with the experimental results, as described in Section 4. In this way, the extracted PSII solution generates hydrogen gas by the light irradiation. These results imply that the extracted PSII solution becomes the fuel for the anode of the fuel cell.

**Figure 8:**
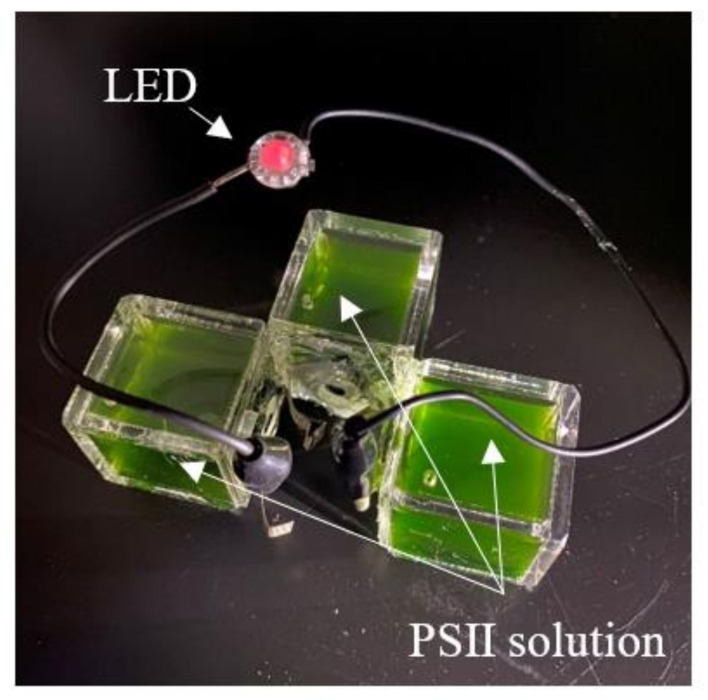
Operation of LED lighting in the PSII biofuel cell.

Figure 8 shows the photograph in which LED is lighted by the PSII biofuel cell. In this experiment, we used the energy source constructed by the series circuit of three PSII biofuel cells with the PSII solution as the fuel. The volume of PSII solution per single fuel cell was adjusted at 20 mL. As shown in Figure 8, we can clearly see that, using three cells, PSII biofuel cells can turn on an LED lamp utilizing the irradiation of white light. This result indicates that the PSII biofuel cell becomes the new energy source using the hydrogen generation of the PSII solution without introducing external hydrogen gas.

**Figure 9:**
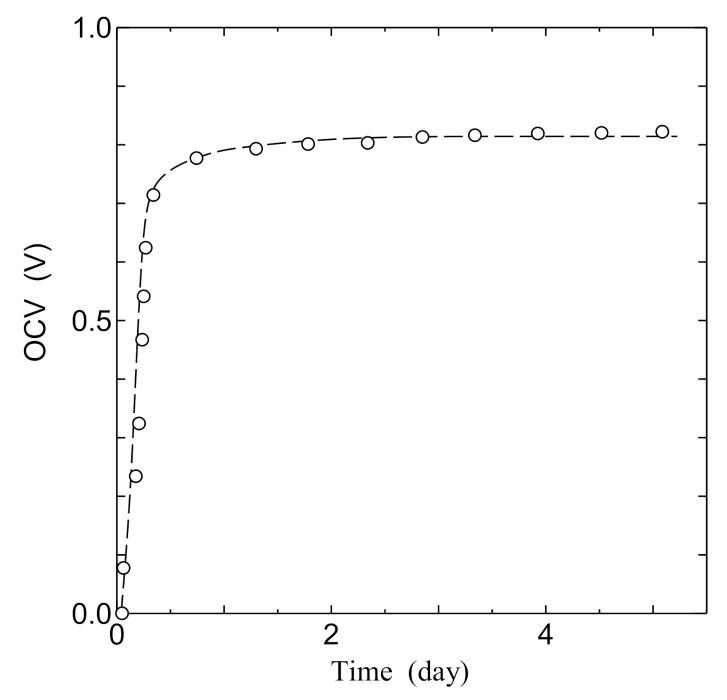
Time dependence of the open-circuit voltage (OCV) in the PSII biofuel cell.

Figure 9 shows the time dependence of the open-circuit voltage (OCV) of the PSII biofuel cell. In this measurement, the PSII biofuel cell was connected with the potentiostat, and OCV was observed with the irradiation of white light measuring 0.5 mW/cm^2^. We can clearly see that the OCV steeply increases with increasing time until one day of irradiation of light, and thereafter becomes almost constant at around 0.8 V.

**Figure 10:**
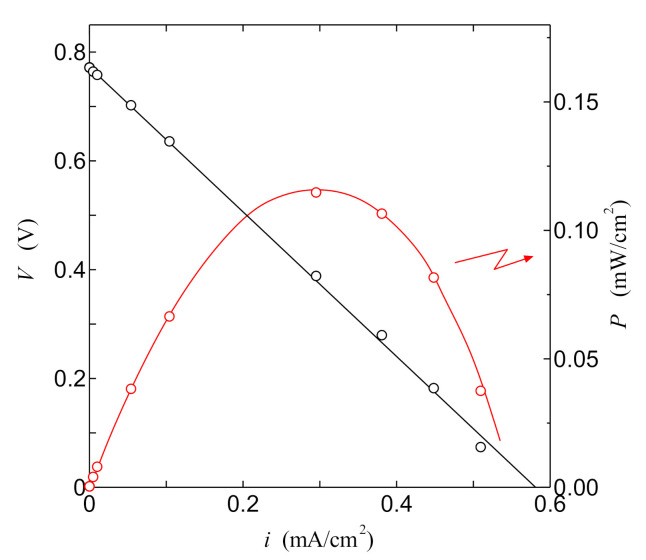
*i–V* characteristics of PSII biofuel cell one days later. Solid line is the theoretical curve.

**Figure 11:**
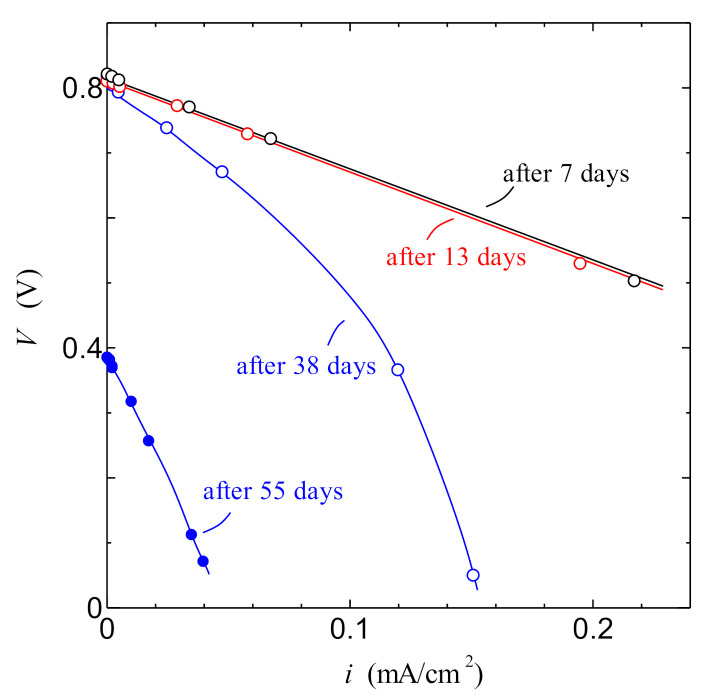
*i–V* characteristics of the PSII biofuel cell over a period of time. Solid lines are the guide to eyes.

Figure 10 shows the relationship between current density *i* and cell voltage *V* in the PSII biofuel cell after one day of irradiation of white light of 0.5 mW/cm^2^. We can clearly see that the PSII biofuel cell shows the typical *i–V* characteristics of the standard fuel cell. This result also indicates that the PSII biofuel cell becomes a new type of fuel cell in the absence of the supply of external hydrogen gas. As shown in the red line in Figure 10, the current density dependence of the power density of the PSII biofuel cell exhibits the parabola curve with a maximum of 0.12 mW/cm^2^. This behavior is also consistent with that of the power density generated by the fuel cell. Figure 11 shows the change over a period of time for the *i*–*V* curve of the PSII biofuel cell. The remarkable change in the *i*–*V* curve cannot be observed until 13 days later. However, the *i*–*V* curve after 38 days shows the anomalous behavior by the lowering of the hydrogen generation by the PSII solution. After 55 days, remarkable decreases in the current density and the open-circuit voltage are observed. These results indicate that the PSII biofuel cell normally operates for 13 days. In addition, if the normal operation of the PSII biofuel cell is required after 14 days or later, it is deduced that the exchange of the PSII solution will be necessary every two weeks.

**Figure 12:**
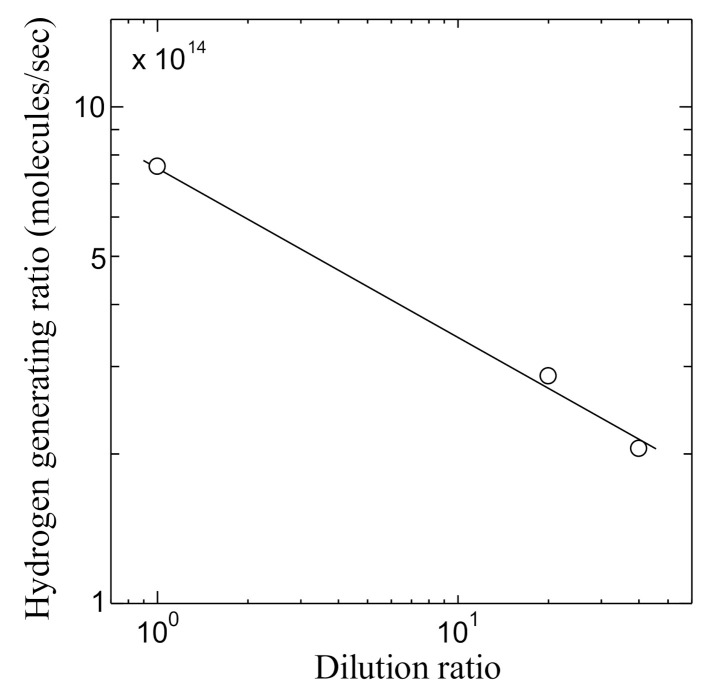
Dilution ratio dependence of hydrogen generating ratio in the PSII biofuel cell.

**Figure 13:**
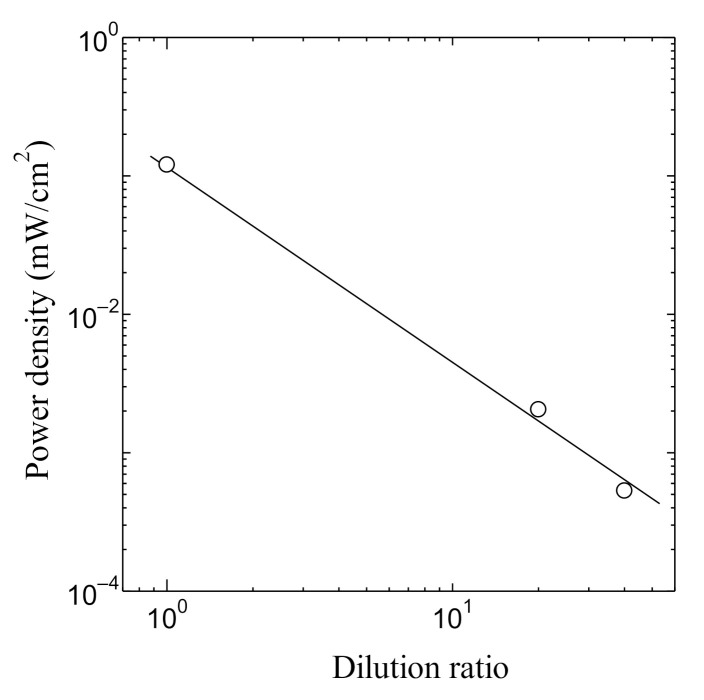
Dilution ratio dependence of power density in the PSII biofuel cell.

Figure 12 shows the relation between the hydrogen generating ratio and the dilution ratio of the PSII solution. As shown in Figure 12, the dilution ratio dependence of the hydrogen generation ratio becomes linear in the log–log plot in all concentrations. In addition, the relation between the dilution ratio and the power density of the PSII biofuel cell is shown in Figure 13. The power density decreases linearly in the log–log scale with the increase of the dilution. Although the results of Figure 12 and Figure 13 are measured independently, the behavior of power density is the same as that of the hydrogen generation ratio. The results of Figure 12 and Figure 13 are consistent with the fact that the decreases of the hydrogen generation ratio lead to the decrease of PSII by the increase in the dilution concentration and results in the decrease of the power density.

## 4. Discussion

The aim of the present study is to report the existence of the new-type fuel cell using the bioelectrolyte using the hydrogens generated by the irradiation to PSII. First, we would like to discuss the consistency of the hydrogen generation by the irradiation to the PSII solution. As shown in Figure 7, the generated hydrogen molecules per second for the irradiation of the red, blue, and white lights were 3.95 × 10^14^, 3.28 × 10^14^, and 7.61 × 10^14^ molecules/s, respectively. It is noted that the sum of the hydrogen numbers generated by blue and red light irradiations is close to those generated by white light irradiation. It is well-known that the blue and red lights are absorbed in the process of photosynthesis [58]. This result is consistent with the fact that the PSII complex mainly absorbs blue and red lights. Considering these facts, the hydrogen generation obtained in the present work is consistent with the result that hydrogen was generated by the photosynthesis reaction.

Next, we would like to discuss the consistency of the open-circuit voltage of the PSII biofuel cell. As shown in Figure 9, the open-circuit voltage (OCV) becomes approximately 0.8 V in the PSII biofuel cell. It is known that OCV of bioelectrolyte fuel cells such as chitin and collagen with the hydrogen fuel gas is typically approximately 0.8 V [29,33]. Considering this fact, the OCV in the PSII biofuel cell is consistent with that in bioelectrolyte fuel cell using hydrogen gas as the fuel gas. This fact also indicates that the appearance of OCV is caused by the hydrogen generation of PSII.

Further, we would like to discuss the consistency from the viewpoint of *i*–*V* characteristics in the PSII biofuel cell. It is well known that the overpotential *V*_ohm_ of the electrolyte lowers the cell voltage *V* of the fuel cell according to theoretical equation *V* = *V*_OCV_ − *V*_ohm_ in the case that the resistance of the fuel-cell electrolyte cannot be neglect, as in the present case. The symbol of *V*_OCV_ denotes the open-circuit voltage, and the value of *V*_OCV_ is 0.78 V from Figure 10. The overpotential of the electrolyte *V*_ohm_ is described by the theoretical equation *V*_ohm_ = *id*/*σ*_DC_ with the thickness of electrolyte *d* and DC proton conductivity of electrolyte *σ*_DC_. Therefore, the cell voltage *V* is proportional to the current density *i*. In Figure 10, the *i*–*V* curve calculated with *V* = *V*_OCV_ − *id*/*σ*_DC_ is shown by a solid line. As shown in the solid line of Figure 10, the theoretical line is in good agreement with the experimental one. Therefore, using the thickness *d* of 135 μm, we can obtain the value of proton conductivity *σ*_DC_ from the slop of the solid line in Figure 10. As a result, the *σ*_DC_ is calculated to be 0.98 × 10^−5^ S/cm. This calculated *σ*_DC_ is in good agreement with the experimental one 0.93 × 10^−5^ S/cm obtained in Figure 3. These results also indicate that the PSII fuel cell is operating by the passing of hydrogen ions via the collagen electrolyte.

In addition, we can estimate the maximum current from the theoretical *i*–*V* curve shown in the solid line in Figure 10. The maximum current is obtained from the value of the maximum current density at the extrapolation of the theoretical *i*–*V* curve to *V* = 0. The maximum current density is 0.59 mA/cm^2^. From this result and the area of electrodes 0.28 cm^2^, we can obtain the maximum current through the anode electrode to be 0.17 mA. As described in Section 3, the calculated maximum current obtained from the Faraday’s second law of electrolysis using the hydrogen generation rate becomes 0.24 mA. This value is close to the experimental value 0.17 mA obtained from the fuel cell performance. Thus, regardless of different experimental methods, the value of maximum current calculated from the hydrogen generation rate is in good agreement with that obtained from the fuel cell performance. These results indicate that the *i–V* characteristics in Figure 10 are caused by the hydrogen generated from the PSII solution.

**Figure 14:**
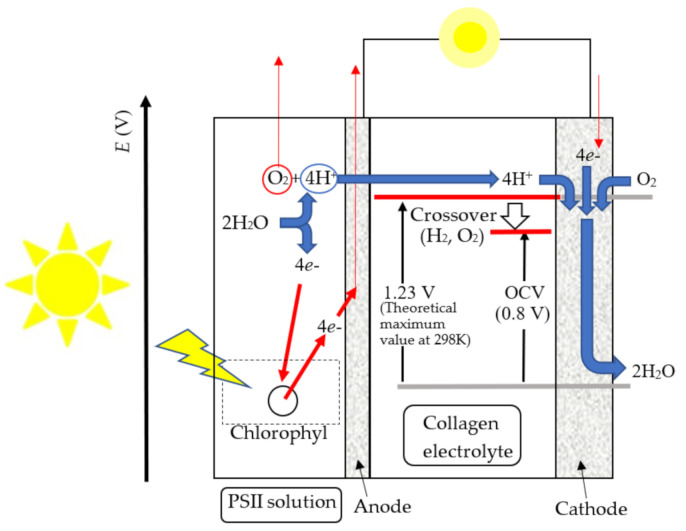
Band diagram of the PSII biofuel cell.

Finally, we would like to show the PSII fuel cell’s operation in the present work using the band diagram of the PSII fuel cell in Figure 14. As shown in Figure 14, the light irradiation excites the electron in chlorophyll and yields the deprotonation of H_2_O by the catalytic reaction of the Mn cluster in PSII. The generated protons are transferred to the cathode through the collagen electrolyte, and the generated oxygen is released in the air. On the cathode, the reaction of 4e^−^ + O_2_ → 2H_2_O is realized. In the present case, the PSII solution generates hydrogens by light irradiation, and hydrogen ions exist on the anode electrode. That is, the PSII fuel cell under the light irradiation operates with the hydrogen-fuel cell mechanism, considering that OCV becomes almost 0 V in the dark. In this situation, the theoretical maximum voltage of the hydrogen fuel cell obtained by generating 1 mole of H_2_O on the cathode is calculated to be 1.23 V from the Gibbs free energy. However, the observed output voltage is reduced by the fuel crossover, such as H_2_ gas transports through the collagen electrolyte, the reaction of other species such as quinones and slight O_2_ cross-reaction in the anode. As a result, in the present PSII fuel cell, OCV becomes 0.8 V under the light irradiation, and we can actually use this voltage in the PSII fuel cell. In this way, the PSII biofuel cell becomes the new-type fuel cell using the biomaterials for the fuel and electrolyte, which are significant parts of the fuel cell. If the PSII solution can be extracted using the disposed leaves and vegetables, energy can be obtained without affecting the environment by combining the fuel cell mechanism and the light irradiation. Therefore, PSII biofuel cells have the potential for next-generation environmentally friendly energy. Now, we plan to investigate the components of the surfactant solution, in order to advance the hydrogen generating rate. These results appear in future issues.

## 5. Conclusions

This study provides the possibility of a new type of biofuel cell. It was found that the biofuel cell based on the collagen electrolyte and the PSII solution becomes a new energy source using the hydrogen by the irradiation of light to the PSII solution. The hydrogen generation rate is 7.61 × 10^14^ molecules/s at the irradiation of 0.50 mW/cm^2^. The PSII biofuel cell exhibits the open-circuit voltage of ~0.8 V and operates LED lighting by the series of three cells. The maximum power density of the PSII biofuel cell is 0.12 mW/cm^2^. The proton conductivity of electrolyte and hydrogen generation rate obtained from the *i*–*V* curve is consistent with those obtained from the theoretical curve. In this way, in the present work, it was indicated that the PSII fuel cell becomes the new-type fuel cell in which biomaterial was used as not only an electrolyte but also as the fuel.
